# When Adenocarcinoma Went Hand in Hand with Neuroendocrine Tumor: A Rare Case of Adenocarcinoma Synchronous with Neuroendocrine Tumor in Ampulla of Vater

**DOI:** 10.7759/cureus.5168

**Published:** 2019-07-18

**Authors:** Tanureet Kochar, Parminder Dhingra, Hamza Shah

**Affiliations:** 1 Internal Medicine, Charleston Area Medical Center / West Virginia University, Charleston, USA; 2 Internal Medicine, St. Vincent Mercy Medical Center, Toledo, USA; 3 Gastroenterology, Charleston Area Medical Center, Charleston, USA

**Keywords:** neuroendocrine tumor (net), synchronous neoplasm, adenocarcinoma, cancer of ampulla of vater

## Abstract

Neuroendocrine tumors (NETs) of gastrointestinal tract are rare entities. Their presence as synchronous lesions with adenocarcinoma has rarely been described in the literature. Cases of synchronous lesions of adenocarcinoma with neuroendocrine component have been described in the colon in the past. However, synchronous presence in the ampulla of Vater is quite uncommon. In the duodenum, NETs constitute 5.7 to 7.9% of the neuroendocrine neoplasms of the gastroenteropancreatic tract. We present a case of 65-year-old male who presented with abdominal symptoms and weight loss, was found to have adenocarcinoma of the ampulla of Vater on biopsy via endoscopic retrograde cholangiopancreatography (ERCP), for which he underwent Whipple’s surgery and was found to have neuroendocrine component along with adenocarcinoma postoperatively on histology.

## Introduction

Neuroendocrine tumors, although rare, account for 0.5% of all the newly diagnosed malignancies [1]. Neuroendocrine tumors have histopathological spectrum ranging from low-grade typical carcinoid to intermediate-grade atypical (malignant) carcinoid to high-grade neuroendocrine carcinomas. Most of these tumors are non-secretory but sometimes they secrete amines, which produces symptoms of flushing, diarrhea, etc.

## Case presentation

**Figure 1:**
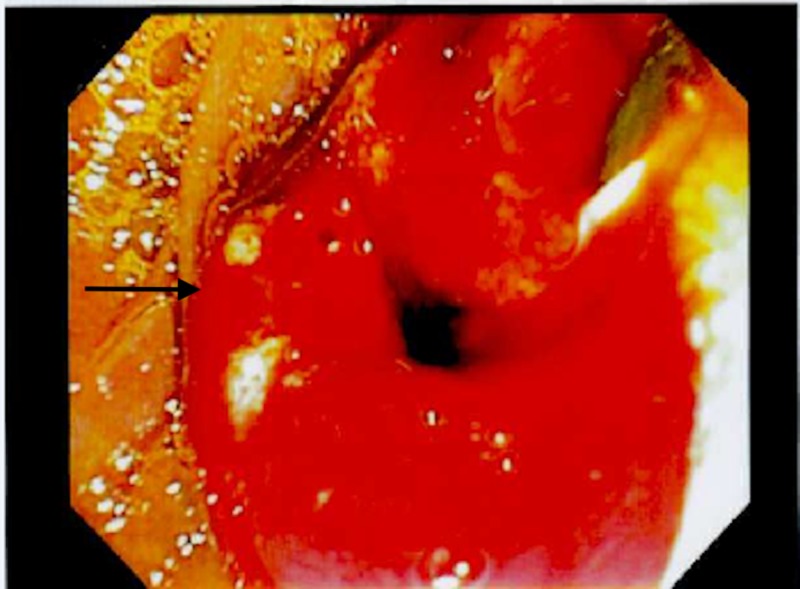
Friable, nodular and ulcerated ampulla

**Figure 2:**
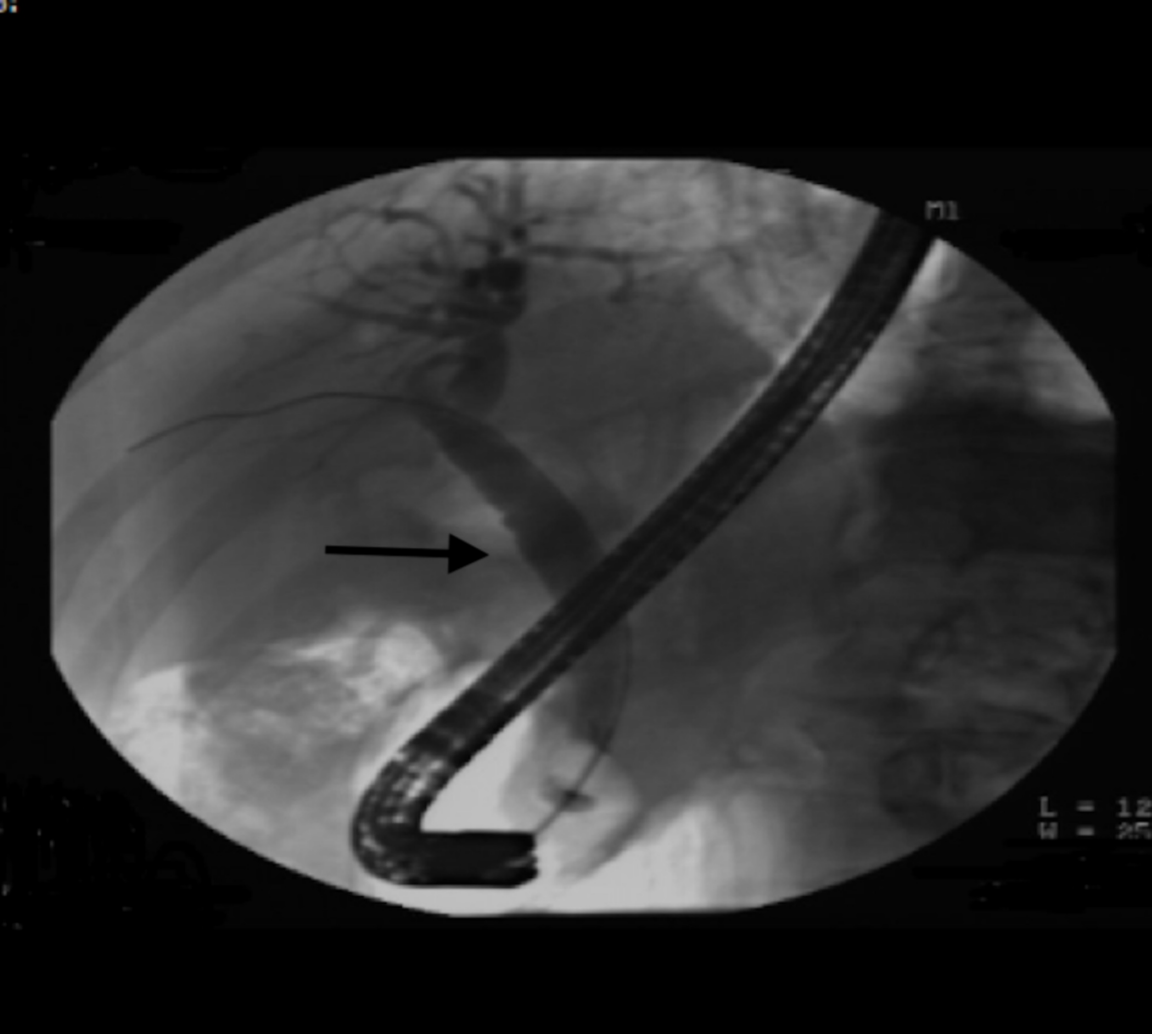
Endoscopic retrograde cholangiopancreatography (ERCP) revealing diffusely dilated bile duct

**Figure 3:**
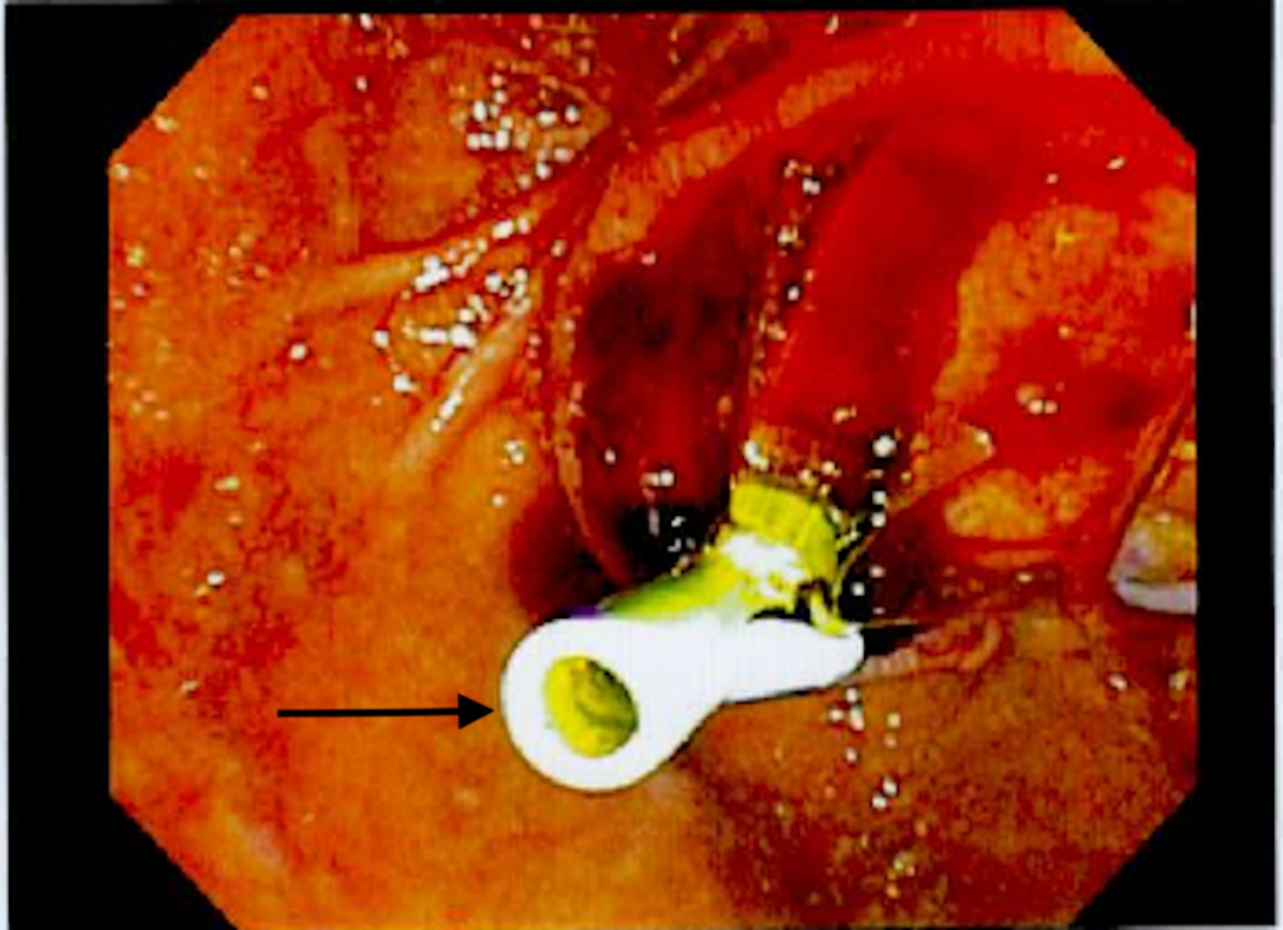
Stent in bile duct

**Figure 4:**
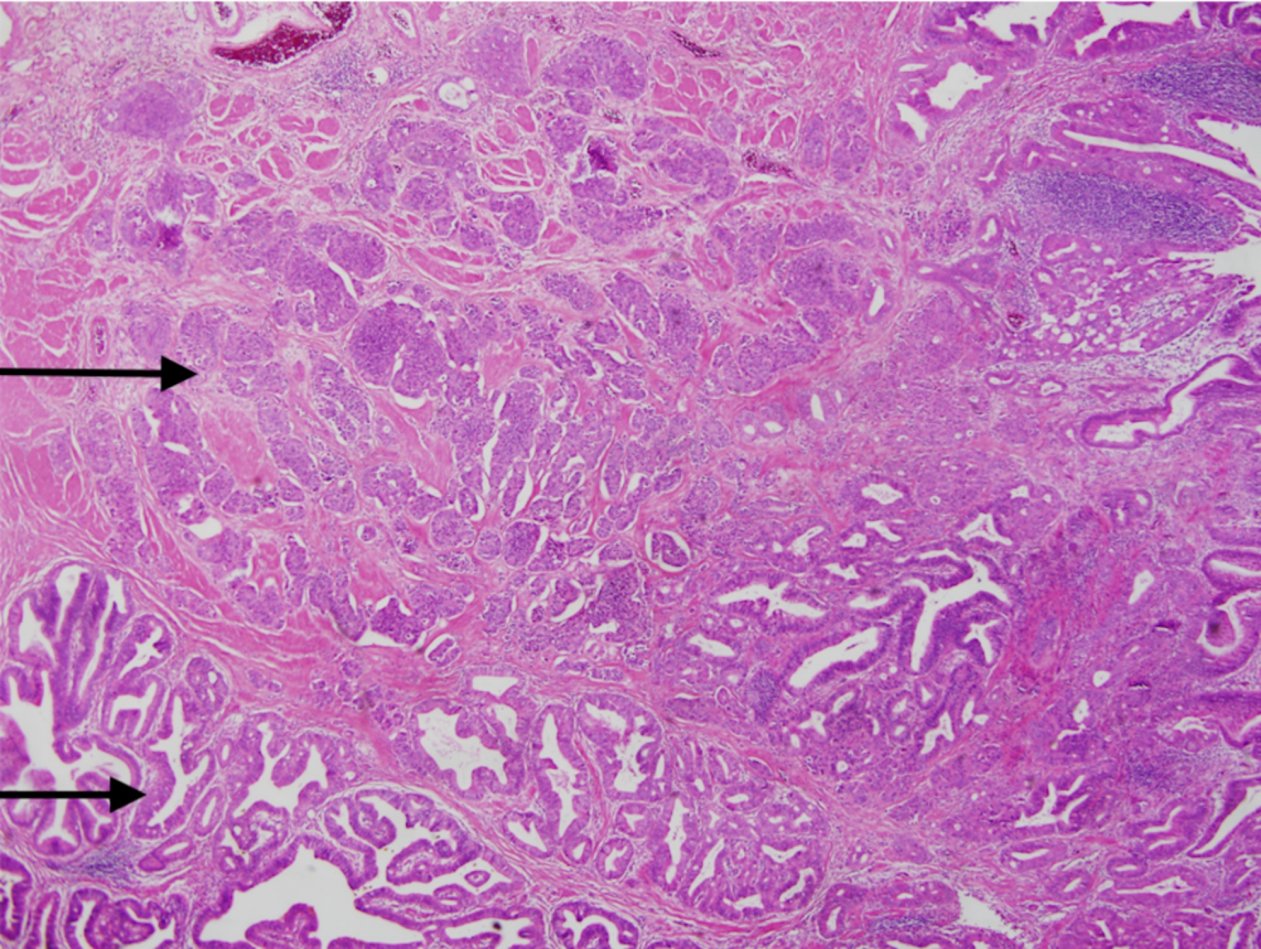
Hematoxylin and eosin (H&E) staining revealing adenocarcinoma and neuroendocrine tumor The top arrow indicates neuroendocrine tumor and the bottom arrow indicates adenocarcinoma.

**Figure 5:**
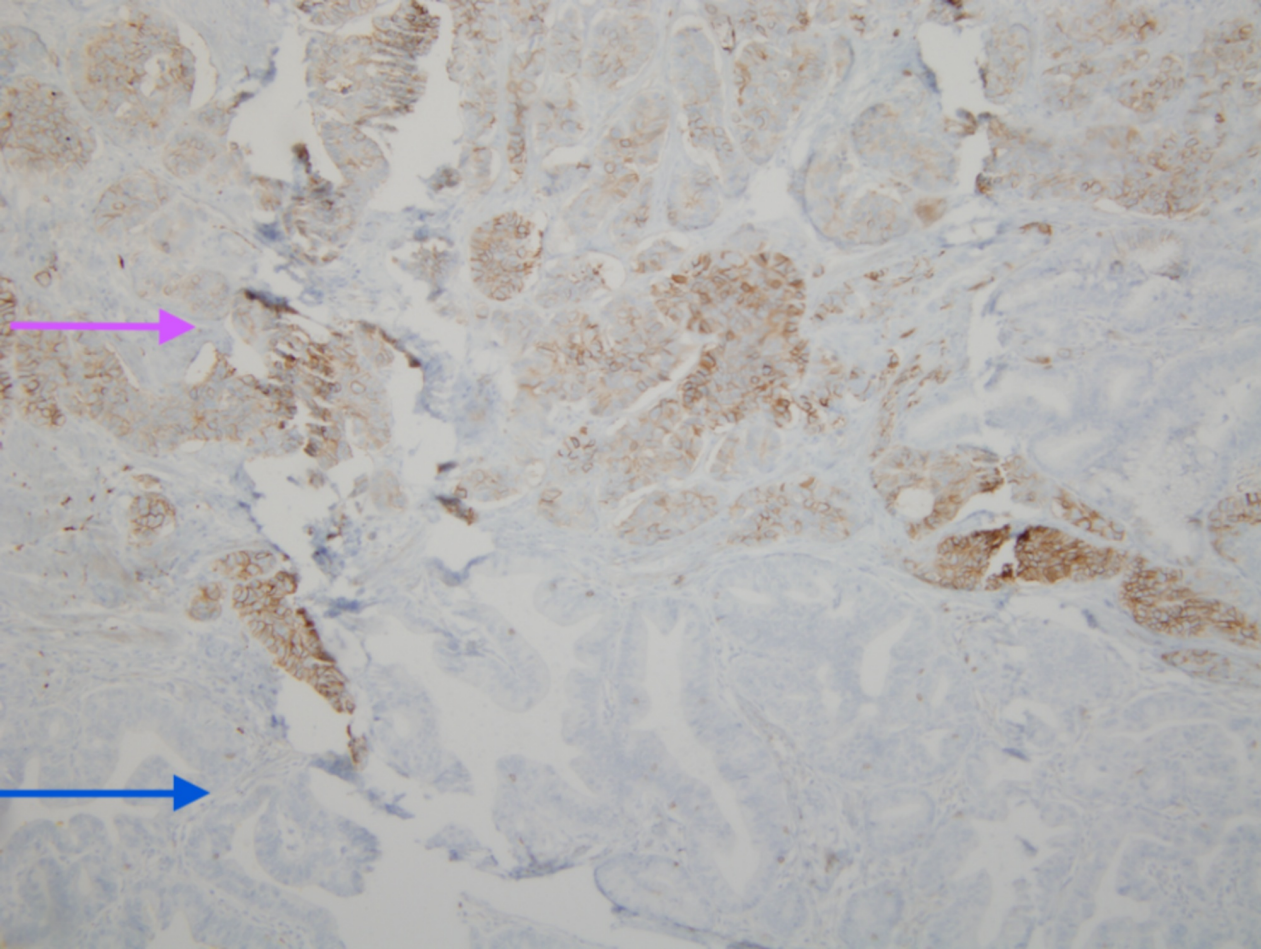
CD56 staining revealing adenocarcinoma and neuroendocrine tumor The pink arrow indicates the neuroendocrine tumor which was stained by CD56 and the blue arrow indicates the adenocarcinoma which was not stained by CD56.

A 65-year-old male with past medical history of Barrett’s esophagus, gastroesophageal reflux disease (GERD), history of (h/o) B cell lymphoma of colon status post (s/p) resection in the past, Parkinson’s disease, hypogammaglobinemia on intravenous immunoglobulin (IVIG) presents with upper abdominal pain, fevers/chills and unintentional weight loss of 10 lbs in the last three months. He also reported pale stools and dark urine. Vitals on presentation were stable. Pertinent labs included complete blood count (CBC) - wnl (within normal limits), basic metabolic panel (BMP) - wnl, albumin 3.4 g/dL, total bilirubin 1 mg/dL, alkaline phosphatase (ALP) 575 U/L, alanine transaminase (ALT) 106 U/L, aspartate transaminase (AST) 115 U/L, amylase 86 U/L, lipase 773 U/L, C-reactive protein (CRP) 10.7 mg/L. He was also found to have elevated tumor markers with carbohydrate antigen (CA) (19-9) 141.2 U/mL, carcinoembryonic antigen (CEA) 5.2 ng/mL. Imaging of the abdomen with CT scan of abdomen/pelvis revealed dilated common bile duct to 1.1 cm and dilated pancreatic duct to 3.8 cm, no obvious mass. He subsequently underwent magnetic resonance cholangiopancreatography (MRCP) of the abdomen which also revealed the similar findings of dilated common bile duct (CBD) with faint signal dropout measuring 2-3 mm at distal CBD. He then underwent endoscopic retrograde cholangiopancreatography (ERCP) which revealed friable, ulcerated and nodular appearing ampulla (Figure 1) with dilated CBD up to 13-14 mm (Figure 2) with distal CBD stricture which was stented with metal stents (Figure 3). Biopsy of the ampulla revealed poorly differentiated adenocarcinoma of ampulla of Vater. Staging computer tomography (CT scan) of chest and abdomen/pelvis was negative for any metastatic disease. The patient underwent Whipple pancreaticoduodenectomy non-pylorus sparing. Biopsy of the distal stomach, duodenum and head of pancreas revealed moderately differentiated adenocarcinoma with 2/8 peripancreatic lymph nodes, although the surgical margins were free of the tumor. It was staged as T2, N1, M0. In addition, he was also found to have poorly differentiated, grade III neuroendocrine carcinoma of ampulla of Vater which was overlaid by ampullary adenoma with no lymph node involvement. It was staged as T1b, N0, M0. The neuroendocrine tumor cells were also positive for CD 56 and exhibited 15 mitosis/10 high power field (HPF) with proliferation fraction of 30% Ki-67 (Figures 4, 5). He subsequently underwent adjuvant chemotherapy with gemtricitabine for four cycles followed by chemotherapy with 5-FU and radiation. He did well with chemotherapy.

## Discussion

The incidence and prevalence of neuroendocrine tumors (NETs) is increasing in recent years due to advanced diagnostic modalities and detection of cancer at early stages. The most common sites of presentation are gastrointestinal tract (62%-67%) and the lung (22%-27%) [2]. In addition, it can involve other organs like ovaries, liver and kidneys. However, among gastroenteropancreatic NET, the ampulla of Vater represents an uncommon site for the disease. According to JAMA Oncology 2017, incidence of NET in gastropancreatic tract is 3.56 per 100,000 [3]. In an analysis of 13,715 neuroendocrine tumors reported over a 50-year period to the Surveillance, Epidemiology, and End Results Program of the National Cancer Institute, only 360 cases involved the duodenum or ampulla [4].

According to few case series, the incidence of secondary primary malignancies (SPM) in patients with gastrointestinal NETs ranged from 12 to 46%, with an average of 17% [5]. Kamp et al. did a retrospective review of 459 patients over a period of nine years and found that the incidence of SPM in patients with gastropancreatic NET is 13.7%, which was similar to what was found in the previous studies [6]. The incidence of SPM with NET is reported to be as high as 55%. In retrospective study in 2015, the incidence of SPM was found to be 15.8% [7].

The synchronous presence of other malignancies associated with NETs is based on field effect theory as is proposed by some authors, according to which the growth of neuroendocrine and other malignancy is stimulated by a common carcinogenic effect. The other school of thought is that NETs secrete various neuropeptides or non-neuropeptides, many of which have specific growth factor properties. For example, gastrin and cholecystokinin (CCK) can stimulate gastric mucosal and pancreatic cell growth [8].

From our literature review, we found only one another case describing the presence of neuroendocrine tumor with adenocarcinoma in ampulla of Vater [9]. Ours is the second case report describing the same. In our case report, NET was indistinguishable from adenocarcinoma macroscopically, however NET was found incidentally by histology postoperatively. We also found two other case reports describing neuroendocrine tumor of ampulla of Vater along with adenocarcinoma of sigmoid colon [10,11].

The modality of treatment depends upon the staging of the tumor, however the principles of treatment remain the same for adenocarcinoma tumor and the neuroendocrine tumor. Pancreaticoduodenectomy is the treatment of choice for adenocarcinoma of ampulla of Vater, which is also the preferred modality for neuroendocrine tumor if lymph node metastasis is present [12,13]. Ampullectomy is reserved only in very selected patients in whom no lymph node involvement is suspected but it is often accompanied by lymph node dissection for complete cure since even in local tumors, the rate of lymph node metastasis is 10% [13,14].

Clearly, the presence of these lesions together in one tumor is not coincidental, therefore suggesting a common link in their pathogenesis. Further studies are needed to determine the pathogenesis of these synchronous lesions. The presence of synchronous and metachronous neuroendocrine tumors with other primary malignancies is being increasingly identified as described above. Further screening with imaging and endoscopy is recommended in patients diagnosed with neuroendocrine tumors to search for primary malignancies.

## Conclusions

The increasing incidence of presence of synchronous lesions with adenocarcinoma is more than merely a coincident. There might be a common link in their pathogenesis which remains unexplored. Further studies are needed to establish this correlation. Since there is increasing incidence of presence of second primary malignancy with neuroendocrine tumors, physicians should screen patients with neuroendocrine tumors to search for primary malignancies.
